# 4E Music Pedagogy and the Principles of Self-Organization

**DOI:** 10.3390/bs8080072

**Published:** 2018-08-09

**Authors:** Andrea Schiavio, Dylan van der Schyff

**Affiliations:** 1 Centre for Systematic Musicology, University of Graz, 8010 Graz, Austria; 2 Department of Music, The University of Sheffield, Sheffield S3 7RD, UK; 3 Faculty of Music, Oxford University, Oxford OX1 1DB, UK; dylan.vanderschyff@music.ox.ac.uk

**Keywords:** 4E cognition, music pedagogy, self-organization, music psychology, interaction theory

## Abstract

Recent approaches in the cognitive and psychological sciences conceive of mind as an Embodied, Embedded, Extended, and Enactive (or 4E) phenomenon. While this has stimulated important discussions and debates across a vast array of disciplines, its principles, applications, and explanatory power have not yet been properly addressed in the domain of musical development. Accordingly, it remains unclear how the cognitive processes involved in the acquisition of musical skills might be understood through the lenses of this approach, and what this might offer for practical areas like music education. To begin filling this gap, the present contribution aims to explore central aspects of music pedagogy through the lenses of 4E cognitive science. By discussing cross-disciplinary research in music, pedagogy, psychology, and philosophy of mind, we will provide novel insights that may help inspire a richer understanding of what musical learning entails. In doing so, we will develop conceptual bridges between the notion of ‘autopoiesis’ (the property of continuous self-regeneration that characterizes living systems) and the emergent dynamics contributing to the flourishing of one’s musical life. This will reveal important continuities between a number of new teaching approaches and principles of self-organization. In conclusion, we will briefly consider how these conceptual tools align with recent work in interactive cognition and collective music pedagogy, promoting the close collaboration of musicians, pedagogues, and cognitive scientists.

## 1. Introduction

A common view in the psychological sciences holds that human cognition is best understood by exploring how individual mental states are determined by their relation with other mental states or with the external world (i.e., behavior and perception). The nature of a mental state, therefore, can be captured by observing its function for the cognitive economy of the system, rather than by its constitutive features. Said differently, a thought or a belief can be realized by an infinite number of diverse physical states, as long as they play the same functional role and instantiate the same causal relations with external stimuli, behavior, or other internal states. This principle has allowed scholars to cross-classify mental phenomena without reducing them to their physical properties, articulating instead generalizations that inform how cognitive architectures are realized in systems that display similar causal predicates. This doctrine, known as ‘functionalism’, has been highly influential for research and theory in cognitive science, artificial intelligence, robotics, and psychology in the last fifty years [1,2]. It promoted the thesis that understanding mental life entails a focus on the theoretical level between physical implementation and behavioral output, emphasizing the explanatory power of mental representations and computations [3,4]. Functionalist psychology, therefore, provided tools and insights that enabled a shift away from the classic Cartesian separation between mind and body. Instead, it offered a perspective where mental and physical states form an interactive, but hierarchical, network based on principles of information processing, where the output functions of the former are specified by the computational possibilities of the latter [5].

The last three decades, however, have witnessed the development of research paradigms that depart from functionalism in a number of important ways. Many of these perspectives fall under the rubric of ‘4E Cognitive Science’ (4ECS)—an umbrella term used to capture the different perspectives associated with the development of a ‘new science of the mind’ [6,7]. Despite the diversity of research contexts associated with 4ECS, scholars working within this orientation are guided by four overlapping principles that understand cognition as fundamentally

- **Embodied:** Cognition cannot be fully described in terms of abstract mental processes (i.e., in terms of representations). Rather, it must involve the entire body of the living system (brain and body).
- **Embedded:** Cognition is not an isolated event separated from the agent’s ecological niche. Instead, it displays layers of co-determination with physical, social, and cultural aspects of the world.
- **Extended:** Cognition is often offloaded into biological beings and non-biological devices to serve a variety of functions that would be impossible (or too difficult) to be achieved by only relying on the agent’s own mental processes.
- **Enactive:** Cognition is conceived of as the set of meaningful relationships determined by an adaptive two-way exchange between the biological and phenomenological complexity of living creatures and the environments they inhabit and actively shape.

Current debates in the philosophy of psychology and cognitive science are heated, and different interpretations of the 4E framework have been offered [8,9,10,11]. Nevertheless, the basic principles of this approach are by now well understood. Because of this, it is becoming a useful model for examining cognitive processes across a range of domains. 

In the present paper, we aim to develop the 4E orientation within the context of music education, showing how this approach can offer a useful way of thinking about pedagogical practice. We explore its descriptive power and consider how a 4E perspective could inspire improvements in educational environments—especially in settings where leftover functionalist assumptions might obscure the contingent and creative nature of musical development and thereby miss the full potential of musical learners. To do this, we will draw on another important concept associated with 4ECS, namely, the notion of ‘autopoiesis’. This was originally developed in the realm of theoretical biology by Francisco Varela and Humberto Maturana [12,13,14,15]. It refers to the self-organizing (or, literally, self-making) and thereby autonomous nature of living organisms. This relates most closely to the ‘enactive’ dimension of 4ECS, where it describes the deep continuity between cognitive and biological processes—between mind and life [16]. As we will discuss in more detail, insights drawn from this approach will allow us to posit that music education environments should be considered as self-organizing and autonomous systems in their own right. It should be noted that our proposal is inspired by existing scholarship in education, music, and improvisation that develops enactive and embodied perspectives on cognition (see, e.g., references [17,18,19,20,21,22,23,24,25]). Our aim is to extend this research and provide a more unitary framework for music education through the lenses of 4ECS, and the notion of autopoiesis. We chose music education as a domain for our music-oriented research for two main reasons. First, it offers an ideal context where theories about social interaction, skills, movements, and experience can be put to the test. Second, recent advances in the field show important similarities and lines of continuity with 4ECS. In particular, the last few decades have witnessed a shift in how music education is practiced and studied—from more traditional pedagogies where a unidirectional stream of knowledge is passed from a teacher to a student, to more relational approaches that highlight improvisation, creativity, collaboration, as well as the role of movement and the situated body for learning [26,27,28,29,30]. We believe this change in perspective resonates closely with post-functionalist cognitive science as it places more emphasis on the embodied, interactive, and adaptive aspects of musicality and human cognition. Accordingly, collaboration between scholars working in both fields could result in insights that are useful to both disciplines (e.g., [31,32]).

The paper is structured as follows. We begin by outlining the notion of ‘autopoiesis’ as it was originally introduced in the biological sciences. Here, we mainly refer to work by Francisco Varela and Humberto Maturana, considering its implications for 4ECS. We then develop these insights in the context of music education, and review some important new perspectives in the field that appear to align with the post-functionalist orientation. Finally, we outline a 4E approach that develops the idea of autopoiesis in the context of music education, showing how this might support and extend the pedagogical approaches discussed previously. While our approach is inherently theoretical and speculative in nature, we believe it may provide important conceptual tools to develop new concrete applications in existing teaching settings. Some examples of this will be explored in the final sections, where the continuity between our proposal and work in social cognition associated with Interaction Theory [33] will also be considered. 

## 2. From Autopoiesis to 4E Cognition

How can we distinguish between living and non-living systems? The Chilean scientists Francisco Varela and Humberto Maturana developed the theory of ‘autopoiesis’ to help draw out this distinction [12,13,14,15]. Put simply, their theory holds that a living system is not a thing (or a finite entity), but rather “a process with the particular property of engendering itself indefinitely” [34] (p. 2). In other words, through circular processes of re-production, the components of a system are constantly generated to maintain the system under precarious conditions [16]. The classic example of this is the prokaryotic cell. As Varela [14] observes, processes of self-regulation maintain the cellular organization as a unity by keeping the thermodynamic relationships with the environment within ranges that are conducive to survival. What lies outside of this biological system does not provide ‘input’ for these processes to unfold: the prokaryotic cell self-produces everything that is needed for its maintenance within its material border, the membrane (see [35,36]). This, however, does not leave the prokaryotic cell in isolation, that is, without a unique relationship with its niche. In fact, a number of processes (e.g., exchange of energy) are realized through the regulatory functions of the membrane itself, which can, for example, modify its permeability when needed (consider here how also the nervous system does not receive information from the environment: it rather “creates a world by defining which configurations of the milieu are stimuli” [35] (p. 15)).

As Mingers [37] notes, it is important to make a clear distinction between the concept of ‘structure’ and the concept of ‘organization’. Maturana defines the latter as the sets of functional relations that determines the system as a unity, while the former is understood as one of the possible manifestations of its interactive components—“thus all cells have the same autopoietic organization, but there are many different cell structures, and a particular cell changes its structure over time” [37] (p. 320). Because these processes unfold at various degrees and timescales, they allow the organism to actively participate in the generation and maintenance of its own survival [38]. The changes in structure of a cell are defined by the cell’s inner norms and not by the perturbations of the external environment, which can only trigger the biological processes the organism will enact to maintain its autopoietic structure. Di Paolo [39] defines this relation with the environment as an “interactional asymmetry”, in which the history of structural coupling between organisms and environments (including other organisms) contributes to maintaining and restoring the organism’s internal balance and its dynamical network of relations with the world. Indeed:

“the self-regulating processes to keep the agent’s conservation as auto-sufficient establish the dialectic between agent and environment: whence the intriguing paradoxicality proper to an autonomous identity: the living system must distinguish itself from its environment, while at the same time maintaining its coupling; this linkage cannot be detached since it is against this very environment from which the organism arises, comes forth”.

[38] (p. 85)

Therefore, as Evan Thompson [16] puts it, “living is a process of sense-making, of bringing forth significance and value” (p. 158). This shift in levels, from biology to cognition, is articulated by Thompson [40] through five points. We report this passage in its entirety:

1. Life = autopoiesis. By this I mean the thesis that the three criteria of autopoiesis—(i) a boundary, containing (ii) a molecular reaction network, that (iii) produces and regenerates itself and the boundary—are necessary and sufficient for the organization of minimal life.
2. Autopoiesis entails emergence of a self. A physical autopoietic system, by virtue of its operational closure, gives rise to an individual or self in the form of a living body, an organism.
3. Emergence of a self entails the emergence of a world. The emergence of a self is also by necessity the emergence of a correlative domain of interactions proper to that self, an Umwelt.
4. Emergence of self and world = sense-making. The organism’s world is the sense it makes of the environment. This world is a place of significance and valence, as a result of the global action of the organism.
5. Sense-making = cognition (perception/action). Sense-making is tantamount to cognition, in the minimal sense of viable sensorimotor conduct. Such conduct is oriented toward and subject to signification and valence. Signification and valence do not pre-exist “out there” but are enacted or constituted by the living being. Living entails sense-making, which equals cognition (pp. 386–387).

The equation of cognition with sense-making thus allows the overcoming of disembodied views about mental life and its properties. It emphasizes the key role of body and action for the realization of cognition and, at the same time, transforms the functionalist focus on computations and hierarchical cognitive structures into a complex network of ongoing relational dynamics between the system’s autonomous agency, its domain of interaction with the world, and the emergence of meaning (as a property central to this network). By this view, sense-making (cognition) cannot be only a matter of representational recoveries from the external world, nor can it be fully described in terms of mental states such as beliefs or thoughts. As useful as those theoretical constructs might be in explaining certain aspects of a cognizer’s high-level mental properties, they arguably downplay a number of important factors: the brain-body system and its relation with the environment, the emergence of meaning through active sensorimotor processes, and the role of interaction with other agents. As we mentioned above, and will discuss in more detail shortly, we can draw important connections with recent work in music education, which increasingly embraces improvisational, informal, relational, and contextual methods of teaching—inherently different from more traditional canons based on knowledge transmission and reproduction of the score (see [41,42,43]). First, however, we would like to further develop the implications of this proposed continuity between sense-making and cognition.

A first consequence of this cognition-as-sense-making orientation is that a body is necessary for mental life—the mind is essentially *embodied*. For ‘body’, we may in fact refer here to ‘two bodies’, as captured by the German words *Körper* and *Leib* (see e.g., [44]). The first describes the objective, physical body—a piece of the world that is measurable and that has an extension. The second term, instead, defines the body as a feeling entity, the living body that we experience in our everyday life. To say that cognition is embodied means that our mental life depends directly on these two (objective and phenomenological) descriptions of the body. It is through our embodiment (which includes the brain) that we develop viable forms of engagements with our world (perception, action, predictions); it allows us to manipulate objects and interact directly with other agents in ways that are meaningful. We do music, for example. We go to concerts, we learn how to play a new piece, we listen to our favorite record, and we move to it. We make sense of the world (including music) through our active participation in its changing demands, enacting musical conducts and non-musical behaviors within environments, as well as inner (metabolic, emotional, etc.) configurations that are appropriate for the context, and that allow for the maintenance of our organizational and structural complexity.

It follows, then, that the explanatory dimension required for the study of cognition cannot be limited to the brain nor to the body. It must go beyond the physical boundaries of the individual because active sense-making entails action, interactions, and other forms of adaptive behavior within the socio-material niche we inhabit and are thus *embedded* in. These elements can only emerge—and be understood—when considering the entire brain-body-world system [45]. Likewise, participating in a musical activity involves social, cultural, and material ecological constraints, whether largely determined by the situation (e.g., a concert at the opera and its ‘rules’) or established as the musical situation develops (e.g., in free improvisations). As such, within the brain-body-world system, cognizers encounter biological and non-biological entities as well as devices that can be helpful in achieving cognitive tasks (e.g., remembering something). And when certain conditions are met, a living system can establish relationships with such factors, resulting in ‘external’ couplings that are relevant to the organism’s well-being as needs and goals are developed contextually [46,47].

This allows for the constitution of hybrid *extended* cognitive systems [48]. For example, consider how biological and non-biological memory (computers) can help us remember an address or a password, or how musical instruments become part of the musician’s cognitive domain such that they can often be experienced as transparent (i.e., as ‘part’ of the performer). Likewise, we might also consider, for example, how an orchestral conductor may offload aspects of his or her cognitive domain into the orchestra, and vice versa. Here rich musically meaningful interactions can emerge, where the agents involved develop novel trajectories of sensorimotor loops (sound, vision, affect, and movement) to achieve the contextual musical goal(s) afforded by the musical event. From this perspective, it is the extended cognitive system involving, in this case, the coupled activities of a conductor, musicians, and instruments (and the audience, the composer, etc.) that constitutes the explanatory field of the musical event.

In being embodied, embedded, and extended, cognition is also *enactive*. This means that the living systems are not simply responders to environments. Rather, they bring forth their own domain of meaning—most fundamentally—through the development of repertoires of actions that are guided by principles related to the organism’s internal coherence (e.g., homeostasis, thermodynamics, regulation, nutrition, reproduction). In doing so, they play an active role in shaping the extended cognitive niche in which they are embedded (the ethical consequences of this approach for musical education are explored in [25]).

In the next section, we will develop these principles within the context of music education. This will allow us to see how such insights can inform and transform concrete pedagogical settings inherent to music, and provide further examples to better capture their relevance for human life more generally. In particular, we will discuss how learning music always entails a bidirectional dynamic where action and perception are coupled, where (musical) meaning emerges through the enactment of concrete, self-organizing, sensorimotor conducts within an extended cognitive ecology, and where, as a result, self-motivated interaction plays a key role in musical action, development, and the flourishing of one’s musical life.

## 3. Steps to a 4E Music Pedagogy

### 3.1. Acquiring Skills, Enacting Meaning

Developing performative skills is an important (and sometimes defining) feature of one’s musical life. But how exactly do we acquire and optimize skills in actual musical contexts? What, so to speak, makes one an expert? One of the most famous models of skill acquisition was developed by Dreyfus and Dreyfus [49], where, to put it in simple terms, learning is thought to occur through a process that begins with the novice theorizing analytically about a certain skill, and that ends with the expert displaying non-reasoned sets of processes that allow him or her to perform the skill ‘intuitively’. A beginner guitar player, in other words, might examine different possibilities for action in a reflective way, trying to remember where to put the fingers, discussing with a teacher the correct position of the right hand, or reasoning about the correspondence between an open string an its sound. Conversely, the expert guitarist has already internalized this knowledge and thus does not need to theorize about a musical situation to accomplish, say, an improvised blues solo: the best way to do a vibrato or the perfect timing for playing the ‘blue notes’ emerge naturally within the contextual flow of his or her musicking. The expert already knows what, or rather, *how* to play in the context: musical directions and decisions, in a sense, are not top-down processes for the expert (similar insights have also been developed by Sudnow [50] in his famous self-reflective phenomenological description of how he learned to improvise with a piano). We should be careful, however, *not* to describe such processes as simply unconscious:

“We should therefore be suspicious of the cognitivist assumption that as we become experts our rules become unconscious. The actual phenomenon suggests that to become experts we must switch from detached rule-following to a more involved and situation-specific way of coping”.

[51] (p. 52); quoted in [52] (p. 52)

As such, the emergence of ‘expertise’ can be described as evolving towards the more holistic, non-reflective, and situated/in-the-moment ability to adapt acquired skills to the context. This ability to engage in forms of ‘skilled coping’ can be found in a range of human activities—athletics, dance, craftsmanship, and more. Likewise, the proficiency of a given musical skill is shown by the agent’s ability to adapt it to the expectations and contingencies of the musical environment being enacted. Consider the following often-cited passage by Dreyfus:

“A phenomenology of skill acquisition confirms that, as one acquires expertise, the acquired know-how is experienced as finer and finer discriminations of situations paired with the appropriate response to each. […]Thus, successful learning and action do not require propositional mental representations. They do not require semantically interpretable brain representations either”.

[53] (p. 367)

While such non-reflective and adaptive forms of skilled coping do indeed appear to be central characteristics of expert performance, the ways novices learn such skills in institutional environments often focus on types of ‘training’ that are highly mediated by rule-following. This is so that students will emerge from such programs with the ability to perform ‘correctly’—i.e., according to standardized modes of (musical) conduct. As Høffding [52] points out, however, this might entail a form of dualism between skilled coping and rule following, where one of the two should be constitutive of our primary awareness. A number of new approaches to musical learning aim to eschew this dichotomy. Here, novices are increasingly invited to participate freely in meaningful musical settings; to explore, create, and situate themselves within contexts associated with non-classical music; to play by ear and to improvise [54,55,56,57,58,59]. Beginners are not asked first to follow abstract rules but are rather immersed in the dynamics of skillful performance starting with their very first musical lesson. We will discuss this in more detail in the following subsection. For now, however, we want to consider two examples from the existing literature that might further illustrate this point.

In the first one [60], a behavioral study comparing memory tasks by pianists, by musicians who did not play the piano, and by non-musicians was reported. Participants from the three groups familiarized themselves with three tonally ambiguous melodies in one of the following learning modalities: (i) actually playing the keyboard, (ii) playing the keyboard with no auditory feedback, (iii) watching a video showing a hand performing the melodies, and (iv) listening to the melodies. The ability to memorize these melodies was measured by testing the participants’ capacity to recognize them among slightly variated versions. The difference across groups, interestingly, was not significant—pianists, musicians who did not play the piano, and non-musicians scored equally. What really mattered, instead, was the learning modality: regardless of their knowledge about music, participants who learned the melodies by playing the keyboard (with or without audio) performed better than the others in their task. This arguably points to a learning mechanism common to both expert and non-experts, one that supports the primacy of what we can call ‘sensorimotor exploration’. Novices and expert musicians need to be motorically engaged with the music to facilitate memorization. There are no sets of explicit rules about music (e.g., recall the stimuli were tonally ambiguous) that were given a priori or that were followed. If rules were generated, these would have been (hypothetically) self-generated by each participant. By physically exploring the sonic environment and by manipulating its properties in ways that are musically meaningful (i.e., playing the keyboard), non-musicians and non-pianists discovered how to make sense of (and memorize) the stimuli being administrated in their own way. Pianists, who had deeper knowledge of music for piano and who were familiar with its set of action possibilities, did not appear to rely on this pre-existing musical knowledge to achieve the task. Otherwise, they would have performed better than the other groups. Accordingly, it might be argued that starting with their first musical experiences, musical learners do not first require sets of organized rules, nor internal models sub-serving decision-making. Rather, they tend to systematically explore the resources of their bodily engagement with the musical environment to optimize their possibilities, and to develop meaningful experiences and understandings (including those related to more ‘high-level’ processes, such as memory or imagination). Similar insights have been noted in the analysis of the first sound-making experiences of infants—where sensorimotor patterns are brought forth to explore and play with the sound properties of the environment they inhabit, resulting in meaningful repertoires of (proto-) musical actions that may be employed in a variety of contextually adaptive ways [61].

Another example we would like to briefly consider involves a recently developed community-based project at the University of Music and Performing Arts of Graz (Austria). Meet4Music (M4M) is an informal pedagogical setting developed around the figure of a facilitator (an expert musician) who guides participants in sessions involving musical and dance improvisation [62,63]. Importantly, the facilitator does not propose a specific set of rules but enables different coordinated possibilities for action and interaction that are explored by each participant in modalities that are meaningful to their personal and cultural backgrounds. Here, the specific boundaries between the participants become fluid and flexible, as they reciprocally adapt to the contingent demands of the musical moments. In the process, the participants collectively enact or, indeed, self-organize their own musical relationships and meanings. The sessions are open to everyone in the community, however a special effort is made to include recent immigrants and refugees. Research has shown that the open-ended and improvisational nature of this program provides a way for established residents and newcomers to interact, and thereby build trust and shared musical goals even when communicating in spoken language is difficult or impossible. Among other things, this program offers a positive response to critiques emerging from recent scholarship in philosophy of music education, which warns of the tendency to downplay musical knowledge and activities other than those associated with the Western canon [64], and how this colonizing bias might promote forms of exclusion. More to the point of this paper, it is also indicative of the ethical implications of an embodied and socially extended approach to music making that takes the principles of self-organization seriously as a central aspect of the fundamentally improvisational and creative nature of living cognitive systems [25,31].

In all, then, the dualistic model of skill acquisition described above appears too static to capture the complexity and the relational dynamics of M4M or to explain the embodied patterns of sensorimotor explorations that novices (and infants) enact to engage with novel musical melodies. The primacy of movement, action, and interaction already defines an open horizon of possibilities rather than a set of rules with which non-experts have to analytically engage. This aligns with the increasing focus put by scholars in educational sciences on interactive, improvisational, and informal kinds of musical learning [65]. Consider, for example, the model developed by Sawyer [30,58,59]. He also starts, like Dreyfus, with the conceptual understanding of a particular technique. Indeed, this requires exploration within a broader context, before being integrated within a more complex set of understandings emerging from practical possibilities. To do so, one needs to be able to generalize from previous knowledge, such that expertise becomes adaptive and flexible in how it responds to the specific situation. Interestingly, however, Sawyer notes that this does not only depend on the inner disposition of the single agent but also on the joint contributions of other individuals who may collaborate and successfully interact to develop the skill in question. The centrality of collaboration for the emergence of musical skills, then, is a fundamental aspect of a 4E music pedagogy. However, such aspects must be cautiously examined. This is because, as we saw in Section 2, there is a specific tension between the inner properties of the organism and its environment, where the latter does not actually determine the inner states of the former. This implies that (i) learning is a modification of the entire brain-body-world system and that, as such, (ii) learning is a self-generating process that is not able to be captured or modified by considering it in terms of an ‘inside/outside’ duality [65] (p. 9).

### 3.2. Autopoietic Musical Learning

Musical learning is a process that does not begin with the individuation of the skill to be acquired from the outside (i.e., from the teacher). This is because, as we argue, a ‘skill’ is not an abstract concept nor an objective feature of musicality that can be easily measured or obtained. Skills are acquired and developed in the sense that they are self-constituted by the entire living organism in its embodied relationship with the environment. By this view, learning is not a finite category but rather a process of indefinite self-production, which maintains the learners in a condition of open realization with their musical environment. We say ‘open realization’ because the various ways musical learners determine themselves are not able to be captured only in terms of how they meet the pre-given requirements of a specific style, genre, or cultural heritage. Beginners must maintain their musical unity—e.g., a meaningful consistency with a specific musical model—through the constant negotiation of their identity. The balance between inner norms, phenomenological requirements, and the fluidity of the musical moment is thus defined by the regulatory functions of the organism enacted within a milieu.

An example may help. Consider a beginner piano player, Leyla. She knows how the piano looks and more or less what she is expected to learn. She knows that her movements will entail a physical engagement with the keys, and that a sound will then be obtained. She also loves the music of Mozart, Bach, and Hindemith. A teacher might help her develop the technique necessary to better explore her potential and to better regulate her dispositions to engage with the piece she is playing. The ‘inner’ norms of Leyla include her cultural and historical background, her identity, her taste for music, her relational dynamics, her behavioral dispositions (e.g., for movement, muscular linkages etc.), her emotional life (e.g., valenced/affective motivations for action, perception, and meaning-making), and so on. Her experience while learning is constantly influenced by such aspects, and the skills to be developed become meaningful through the constant integration of various overlapping aspects of these dimensions. Leyla’s metabolic and emotional responses to the learning process are determined by such relationships, for it is only through the unification of these components into various self-organized configurations that Leyla can generate new musical understandings and possibilities (i.e., skills) that may be re-used, adapted, and further developed.

In other words, Leyla’s biological organization enacts a self-generated ecology for learning, whereby different components configure and calibrate themselves in relation to each other and to the musical environment in which Leyla participates. A Mozart piano sonata can be learned only when certain conditions within this complex nonlinear network of states and functions are met. However, we do not think that, on the most fundamental levels, such conditions necessarily involve the generation of concepts [10] or the adherence to rules. Instead, we claim Leyla’s cognitive ecology organizes itself first and foremost through processes of sensorimotor exploration involving the interplay between the structural organization of her body, the dynamics and affordances of the environment, and the emotional/affective processes associated with regulating her corporeal states. In doing so, skill emerges through the significances and valences that are enacted in each musical action. Exploring a musical world, as Leyla does every time she plays the piano, is a manifestation of the global state of the system, which seeks for a stable relational domain by constantly shifting goals, intentions, relations, emotions, and motivations. Accordingly, learning is best understood as a process where the entire brain-body-world network dynamically changes itself. Yet, these changes are not simply defined by the shifting musical landscapes that Leyla creates and is exposed to—these can only trigger responses. A change in the network depends on the biological and phenomenological norms inherent to the system itself. From here it follows that the entire system participates constitutively in the development of musical skills, such that elements of the body, the world, and the brain constantly develop new states and configurations to define the learning trajectories Leyla experiences.

4ECS can help us better capture the structural and organizational dynamics central to such an interplay. Let us first consider the role of the body. The body-in-action becomes the ultimate source for meaning ascription in the learning process. Consider how a cello player uses her body to facilitate certain aspects of her performance of a scale and uses her bodily proportions (e.g., the relationship between the cello, her hand, and her arm in a given position) to remember the correct fingering. Or imagine a novice drummer learning how to play a newly acquired double bass drum pedal. The way the body feels the pedals, the position of the drummer’s back and seat, and then the way the drums respond in feeling and sound to the bodily action as integrated with the pedals are all part of the cognitive ecology constituting the learning process. Any set of rules the learner might encounter in the process—e.g., those imposed (or suggested) by a teacher or by the learner herself—will need to be incorporated in terms of bodily knowledge to drive the learning process successfully. This transforms the conception and function of ‘rules’ into fluid entities that stimulate explorations and new discoveries (rather than fixed categories to be either respected or violated). This does not mean that teachers should not intervene, discuss, suggest, and facilitate the student in achieving a particular musical goal. A repertoire of options and open possibilities to perform a scale (or a set of suggestions about playing the double bass pedals) is certainly an important resource for beginners to familiarize themselves with the instrument and its relationship to the body—learning technique is important and highly useful. However, there is an important difference between fostering self-generative forms of discovery and more prescriptive forms of exposure. By including the kinds of open-ended, exploratory, embodied, and improvisational action with instruments and sound that learners naturally tend to produce [32], an open horizon of relationships and possibilities is disclosed and enacted: the body does, the body feels, the body predicts and anticipates; it has certain layers of autonomy that can lead to new meaningful interactivities with the musical instrument, without involving mental plans, rules, or normative domains [66,67,68,69].

If the body plays a key role in determining musical learning, so does the socio-material and cultural environment in which it is embedded. However, these dimensions are not separated from the body. Rather, they become manifest through the body and are co-determined by actions and interactions with other bodies and things in socio-culturally meaningful contexts [47]. It follows, then, that if learning is a self-producing activity involving the development and adaptation of patterns of sensorimotor engagements that allow us to make sense of the world we inhabit, then the sociocultural environment that we live through and that we play an active role in shaping will have to be taken into serious consideration when developing any pedagogical strategy. For example, consider how a beginner rock guitarist might learn a solo by watching a video of her favorite guitarist. What strategies will she adopt to imitate the solo and (at the same time) develop her own musicianship? There is a sense here in which the notion of self-imposed rules becomes either too general (e.g., she will follow the rule of imitating the movement of the guitarist’s fingers) or too strict (e.g., she will systematically select the motor programs adequate for the given musical phrase through the analysis of different parameters from the video, her instrument, and her bodily posture). In both cases, however, motivated sensorimotor explorations are hindered. She will probably begin by exploring the instrument, experience how it feels in a certain way, try out different phrasing possibilities. Aspects of her musical identity, cultural understanding, and musical taste are engaged and developed through such explorations, informing how it will unfold. Does this distortion pedal make the sound more similar to a style she does not like? Or does it make it sound more in line with the guitarist she wants to imitate? The instrument itself offers possibilities for learning that can be discovered and experienced through action. Sensorimotor exploration allows the guitarist to gain familiarity with the instrument and the music through non-systematic configurations of the entire system, as its global state is determined by variables the learner may not be aware of (e.g., the structure of the musical phrase, the option to use a different fingering, etc.). Indeed, in the process, the guitarist might discover possibilities for sound and action that go beyond imitation. And this might lead to the development of original musical creations that push against or advance how and what rock guitar playing entails, for the learner and perhaps even for the broader socio-cultural milieu (e.g., see the discussion of Coltrane and Hendrix in [70]).

Additionally, as we have suggested above, the self-generating properties associated with musical learning also involve the ways tools and devices from the environment are coupled to the entire system. This allows the creation of hybrid extended cognitive systems where musical instruments, scores, and technological devices are often exploited in novel and sophisticated ways. Such encounters also shape the learning trajectory of a novice. We have touched on this above in our discussion of the guitarist who, as she develops greater proficiency, is able to offload various musical tasks to the instrument. These patterns of activity are only effectively recalled in direct interaction with the object (the inclusion of delay effects, sampling, or sequencing software takes this a step further). This can also be understood in terms of interpersonal learning. The concept of autopoiesis associated with 4ECS can thus be helpful to describe and interpret a number of music-specific practices. Consider how a developing ensemble must learn to adaptively take on and offload various tasks to each other—providing or entraining with a rhythmic pulse, leading and following phrasing and intonation, or constructing improvisations. Even when the music they are performing involves a clear leader-follower schema, a closer analysis might show how the follower is in fact an integral, constitutive part of the leader and vice versa. Again, the entire musical system self-organizes and modifies its own structural components to reach its optimal configuration (e.g., a leading internal voice that is clearly distinguished from the ensemble, but that is nevertheless supported and given meaning and context by the background texture). This idea has implications for pedagogy, as it gives equal status to the components that do not immediately manifest as playing leading roles for the musical event. Accordingly, it can inspire students and teachers to further explore ‘hidden’ (e.g., relational or structural) aspects of a score, create novel ways to learn a scale more musically, or interact and freely improvise, beginning with timbral, rather than with melodic or harmonic material.

Similarly, one might also consider how a composition student may gain relevant knowledge of the harp by asking a professional harp player about its main features (e.g., the double-action pedals). The developing composer gains understandings of the possibilities of an instrument she does not play by interacting with the harpist as an extended cognitive resource. Likewise, when the harpist prepares and performs the piece, she too becomes part of an extended cognitive network, including the composer, instrument, the audience, and the other musicians. As we considered above, sets of rules and norms associated with traditional practice could initially inform such learning processes. However, these need not be understood as prescriptive. For example, harps and guitars can be prepared, detuned, or played in unconventional ways; ensembles can experiment with various configurations and approaches. Thus, norms and rules might be conceived of as transitory affordances that can stimulate further discoveries and engagements with musical instruments (or other objects), and other people. Again, this has concrete implications for music pedagogy: novel ways of interacting with a musical instrument can be developed, explored, and enacted. Here, the self-producing and regenerating resources adopted to develop such new agent-instrument interactivities extend the operational possibilities of the living system, allowing the emergence of a novel musical world. Indeed, through the structural reconfiguration of the brain-body-world coupling, the global action of the learner entails an open horizon of musically meaningful conducts, one where learner and learning become two aspects of the same autopoietic process. In addition, because of the seemingly paradoxical openness of such self-organizing networks—which, on one hand co-emerge with(in) the environment, and, on the other hand, maintain their autonomous identity—different structural couplings with the socio-material environment can entail a variety of strategies, options, and possibilities that are hardly reducible to a list of instructions. For this reason, the possibility of open communication between the learning environment and the developing student represents a vital resource for the latter’s flourishing as a musical agent. This also leads us to consider the different impacts formal (more prescriptive) and non-formal music (less prescriptive) environments can have on the internal balance of the autopoietic network in development (i.e., the learning musician) and how these lead to different learning trajectories and outcomes. We suggest that this understanding of learning as continuous with the self-organizing properties of living beings might enrich our knowledge of music pedagogy and support calls for the inclusion of more open-ended and creative environments for music education.

## 4. Conclusions

We have considered here how the processes involved in musical learning may be seen as continuous with (but not simply reducible to) the basic bio-cognitive dynamics by which all living systems bring themselves into existence and strive to maintain a flourishing lifeworld. In doing so, we have explored musical learning in terms of the adaptive, embodied, interactive, and goal-directed processes by which autonomous agents enact repertoires of action and perception that enable coherent relationships between the ‘self’ and the contingent socio-material environments they inhabit. This has involved the introduction of a number of concepts that could be useful to teachers and learners in guiding development—most centrally the idea of autopoiesis and the 4E framework. Importantly, the orientation we have developed here highlights the self-organizing nature of learning and therefore has certain bio-ethical implications for pedagogy. Indeed, if the processes and dimensions we have described here define the characteristics of what it means to be a living agent, then they should be understood and properly encouraged in music education. Thus, while our aim here has not been to tell teachers what and how to teach, the perspective we have introduced could offer some general principles for guiding future research and practice.

Above all, this approach asks teachers and students enact pedagogical environments where they may exercise their capacities as self-making musical beings—where exploratory, improvisational, creative, and collaborative activities can play out. As such, an ethics of care [71] is considered as a fundamental element to be developed in these contexts, one that involves a relational contextualization of a musical learner and her needs to encourage the natural development of her learning trajectory. Additionally, because students do not learn in isolation, this orientation also involves a greater appreciation of how social understandings arise. Here, recent work associated with Interaction Theory (IT) offers some promising possibilities [72,73]. Put simply, this approach considers social cognition not only in terms of how agents internally represent, simulate, or theorize about the mental states of others. Instead, empathic processes are explored first in terms of embodied affective interactivity that allows for shared understandings to develop between autonomous agents [74,75,76]. Similar perspectives have only just begun to be explored in musical contexts [77,78], and we look forward to seeing what new insights it offers, especially since it also develops the 4ECS orientation discussed above.

Lastly, we would like to note that the embodied, embedded, extended, and enactive aspects of the 4E framework also offer useful dimensions by which teachers, students, and researchers could begin to explore and assess the processes involved in musical learning. For educators, an autopoietic teaching strategy might involve the individuation of the specific musical goals a student aspires to achieve. This can be followed by an open discussion where these goals are analyzed in light of other non-musical parameters (e.g., cultural background) and developed together through shared forms of musicking. In a sense, the additional non-musical parameter might act as a perturbation, which can trigger structural changes in how the initial goal is perceived and obtained. It is expected that such a shift in the stability of the students’ learning trajectory could motivate them to explore and develop novel musical possibilities (e.g., new technological devices, sound-making options with their instrument, new ensemble configurations, or a new posture while performing). Indeed, as an autonomous being immersed in a cultural and musical niche, a student’s coupling with the environment cannot be limited only to the desired musical goal. Instead, it must also involve the whole complexity of its phenomenology—a vast array of being, knowing, and doing, which are co-constituted by the learner, the teacher, the environment, and their coupling (see reference [25] for discussion). The self-organizing capacities of the learner, in other words, are best understood when considering the development of novel and meaningful patters of action and perception that continuously shape this structural network, involving different levels, timescales, and (social) interactivities. As such, we suggest that richer forms of learning may be realized through a shift in focus from stable pre-given ‘skills’ to be acquired to a more open horizon of less predictable and exploratory possibilities that emerge and develop constantly: a self-organizing process in which agents strive to maintain their adaptive stability and flourish as autonomous musical beings. How to navigate the complexity of such a dynamic network of musical, social, and cultural parameters (which are always to be considered as co-emerging from each other, rather than objectively individual), remains an open empirical question that has been posed in different domains. Indeed, various possibilities for research are beginning to be considered in the context of music and human evolution [79], musical development in infancy [61], musical creativity [70,80,81], musical emotions [82,83,84], improvisation [85], music therapy [67], and more. In all, the principles of bio-cognitive self-organization and 4ECS appear to have a great deal to offer to emerging pedagogies that aim to better understand and encourage the creative and world-making possibilities of musical learners.
